# Techniques for Food Authentication: Trends and Emerging Approaches

**DOI:** 10.3390/foods12061134

**Published:** 2023-03-08

**Authors:** Margit Cichna-Markl, Isabel Mafra

**Affiliations:** 1 Department of Analytical Chemistry, Faculty of Chemistry, University of Vienna, Währinger Straße 38, 1090 Vienna, Austria; 2 REQUIMTE-LAQV, Faculdade de Farmácia, Universidade do Porto, Rua de Jorge Viterbo Ferreira, 228, 4050-313 Porto, Portugal

Food producers and retailers are obliged to provide correct food information to consumers; however, despite national and international legislation, food labels frequently contain false or misleading statements regarding food composition, quality, geographic origin, and/or processing. Food authentication is very challenging, requiring highly selective, sensitive, accurate, reproducible, and robust analytical methods. This Special Issue of *Foods*, comprising ten research and two review articles, highlights recent advances in food authentication and clearly demonstrates that no single method is suitable for covering all aspects of food authenticity.

Undoubtedly, DNA-based methods that target nuclear or mitochondrial (mt) markers have played a key role in the identification and differentiation of species and/or cultivars in food. Real-time PCR continues to be the technique of choice for the authentication of diversified food commodities, owing to its high specificity, sensitivity, and reproducibility. This is also the case for species authentication in meat products, for which real-time PCR has been one of the most widely applied DNA-based techniques, mainly targeting mtDNA [1]; however, the quantification of meat species, or any other food, using real-time PCR is challenged by the accurate preparation of reference mixtures as calibrants for method development. The choice of DNA marker is also challenging, especially when the purpose is quantitative analysis. Despite the advantages of mtDNA regarding sensitivity and specificity, its variable copy number is a drawback for quantitative approaches. Accordingly, a TaqMan real-time PCR assay targeting the lactoferrin gene of roe deer was developed for its quantitative determination in meat products [2]. The assay was validated in-house by determining the roe deer content in model meat mixtures and a model sausage, after which it was applied to the analysis of commercial meat products [2]. Nevertheless, method standardization requires assessment through interlaboratory trials [3]. Therefore, the real-time PCR assay for roe deer was tested in an interlaboratory ring trial, including 14 laboratories from Austria, Germany, and Switzerland. The assay proved its applicability to detect and quantify roe deer in raw meat samples to detect food adulteration, though further trials are still needed to validate its application to thermally treated model foods [4].

The application of real-time PCR was also demonstrated in plant species authentication in a particularly challenging matrix—vegetable oil. For the first time, new qualitative and quantitative PCR assays were proposed to authenticate argan oil [5]. Argan oil is a premium product, commercialized worldwide as cosmetic- and food-grade, which is potentially adulterated with other vegetable oils. To address this problem, two real-time PCR calibration models were developed by using the normalized ΔCq method to estimate potential adulterations of argan oil with olive or soybean oils, after which it was validated in-house with blind mixtures [5].

DNA barcoding targeting the cytochrome c oxidase subunit I (COI) gene, as a relatively conserved region with sufficient variation among species, has been widely applied in the species authentication of seafood [6]. COI barcoding was employed to analyze sushi samples collected by means of the citizen science approach, involving people from eighteen different Italian cities (Northern, Central, and Southern Italy). Data indicated a substantial rate of species substitution—between 31.8% in Northern Italy and 40% in Central Italy. *Thunnus thynnus* was the species most frequently replaced, followed by flying fish roe substituted with the eggs of *Mallotus villosus* [7]. DNA metabarcoding, a combination of DNA barcoding with next-generation sequencing (NGS), is an emerging approach that allows for the detection of multiple species in complex and processed foods, overcoming the drawbacks of Sanger sequencing [6]. Bivalve species belonging to the Mytilidae (mussels), Pectinidae (scallops), and Ostreidae (oysters) families were successfully identified in foodstuffs via DNA metabarcoding using fragments as small as 150 bp of mitochondrial 16S rDNA [8]. The feasibility of DNA metabarcoding using 16S rDNA was also demonstrated in an analysis of mammalian and poultry species in food and pet food [9].

The varietal authentication of foods is another challenging task that has been successfully overcome by using DNA markers, namely microsatellites or simple sequence repeats (SSR). This was demonstrated in the case of olive species. Microsatellite markers have been employed in cultivar identification, characterization of autochthonous olives (ancient olive trees and oleasters), management of olive germplasm banks, phylogenetics, diversity analysis, and mapping, as reviewed by Yadav et al. [10].

In recent years, several studies have demonstrated the feasibility of using spectroscopy for food authentication purposes, taking advantage of its non-destructive character, simple sample preparation, and possibility of being operated by non-expert technicians. Nevertheless, the effective application of spectroscopic approaches in food authentication relies on the construction of suitable spectral databases and multivariate analysis. The combined techniques of Raman, near-infrared (NIR), and fluorescence spectroscopy were applied to the analysis of chia oils adulterated with sunflower oil [11]. Fourier transform mid-infrared spectroscopy with attenuated total reflection (ATR-FTMIR), coupled to multivariate analysis, was applied to discriminate doughs and 3D-printed baked snacks, enriched with edible insect (*Alphitobius diaperinus* and *Locusta migratoria*) powders [12].

Chromatographic techniques, particularly those hyphenated with mass spectrometry (MS), have provided some of the most powerful tools for the detection and identification of chemical markers for food authentication. Aiming at authenticating hay milk, a traditional dairy product recently launched on the market and protected as “traditional specialty guaranteed” (TSG), two chromatographic techniques were proposed. Gas chromatography with MS detection was used as a targeted approach to detect the cyclopropane fatty acid (dihydrosterculic acid, DHSA), a marker of the bacterial strains found in silage since hay milk should be obtained from a cow’s feed ration free from silage. The detection of DHSA could be related to the presence of maize silage in feed, though it was ambiguous in the case of grass silage [13]. High-performance liquid chromatography (HPLC) coupled to high-resolution mass spectrometry (HRMS) was then used as an untargeted approach to characterize the lipidic profile, resulting in the identification of 14 triacylglycerol biomarkers in milk. The biomarker profiles, combined with a multivariate analysis, allowed to predict the use of maize and grass silage in a cow’s diet with 100% recognition [13]. Comprehensive two-dimensional gas chromatography (GC×GC) is an advanced approach with high potential to characterize complex volatile fractions of foods, including enantiomeric recognition for authentication purposes, owing to its improved separation capacity, increased number of identified compounds, structured chromatograms, and significant signal enhancement [14]. Vyviurska et al. [15] exploited an enantioselective GC×GC analysis to assess botrytized wines in comparison to the corresponding varietal grape wines, selected essences, and varietal wines fermented with grape skins. After a hierarchic cluster analysis, the data showed that the varietal wines were successfully separated from the other types, and a correlation between the botrytized wines and the varietal wines fermented with grape skins could be observed [15].

The multi-element composition of an animal’s tissues can reflect, to some extent, its diet, while in the case of plants it reflects the soil composition of the location where they grow. Therefore, direct information about the geographical origin of foods can be provided by the bioavailable nutrients underlying the soils [16]. Conversely, elements found in the tissues of aquatic animals, such as fish, are recognized as being derived from the elemental composition of the overall surrounding environment, the aquatic habitat to the production premises, which is particularly useful when intending to identify the country of origin of wild specimens [17]. The application of element profiling approaches to fish and seafood products has been gaining force due to the advances in the optimization of existing instrumentations for multi-elemental analysis, but also due to improved algorithms for statistical analysis. From the review of Varrà et al. [17], the discrimination of geographical origin has been the most frequently reported authenticity topic, while other aspects, such as farming systems, have been overlooked. From the available methodologies, inductively coupled plasma mass spectrometry (ICP-MS) for elemental speciation and ICP-MS/MS for interference-free determination as well as isotope ratio measurement are anticipated as turning points for the high-throughput analytical characterization of complex matrices, such as food [17]. Accordingly, the multi-elemental profiles of 237 walnut samples from 10 countries and 3 years of harvest were analyzed via ICP-MS, and the data were evaluated with chemometrics, including machine learning methods. The results showed that walnut cultivar and harvest year had no observable influence on origin differentiation and highlighted the high potential of element profiling for the origin authentication of walnuts [18].
